# Prospective association of device‐based physical activity and sedentary time during childhood with mental health outcomes during adolescence

**DOI:** 10.1111/camh.70048

**Published:** 2025-12-02

**Authors:** André O. Werneck, Chih‐Sung Liang, Lee Smith, Dara Aldisi, Nasser Al‐Daghri, Arnold Baca, Liye Zou, Qian Yu, Jin Kuang, Zhihao Zhang, Marco Solmi, Brendon Stubbs

**Affiliations:** ^1^ Center for Epidemiological Research in Nutrition and Health Universidade de São Paulo (USP) São Paulo SP Brazil; ^2^ Department of Psychological Medicine Institute of Psychiatry, Psychology and Neuroscience, King's College London London UK; ^3^ Department of Psychiatry Tri‐Service General Hospital, National Defense Medical Centre Taipei Taiwan; ^4^ Department of Psychiatry, Beitou Branch Tri‐Service General Hospital Taipei Taiwan; ^5^ Centre for Health Performance and Wellbeing Anglia Ruskin University Cambridge UK; ^6^ Department of Community Health Sciences College of Applied Medical Sciences, King Saud University Riyadh Saudi Arabia; ^7^ Chair for Biomarkers of Chronic Diseases, Department of Biochemistry College of Science, King Saud University Riyadh Saudi Arabia; ^8^ Centre for Sport Science and University Sports, University of Vienna Vienna Austria; ^9^ Body‐Brain‐Mind Laboratory, School of Psychology Shenzhen University Shenzhen China; ^10^ School of Epidemiology and Public Health, Faculty of Medicine University of Ottawa Ottawa Ontario Canada; ^11^ Department of Child and Adolescent Psychiatry Charité Universitätsmedizin Berlin Germany; ^12^ Department of Mental Health The Ottawa Hospital Ottawa Ontario Canada; ^13^ Department of Psychiatry University of Ottawa Ottawa Ontario Canada

**Keywords:** Exercise, mental health, sitting time, movement behaviors Youth, adolescence

## Abstract

**Background:**

The present study aimed to analyze associations between device‐measured physical activity and sedentary time during childhood with mental health‐related outcomes throughout adolescence.

**Methods:**

Data from the Millennium Cohort Study were used. Device‐measured physical activity (i.e., light and moderate‐to‐vigorous physical activity) and sedentary time were assessed at 7 years. Internalizing and externalizing symptoms were assessed at three time points (11 years, 14 years, and 17 years) via the Strengths and Difficulties Questionnaire. Depressive symptoms were estimated using the Mood and Feelings Questionnaire at 14 years. Psychological distress was assessed using the six‐item Kessler psychological distress scale at 17 years. Negative binomial regression models, adjusting for potential confounders were used.

**Results:**

Among boys, greater time spent in moderate‐to‐vigorous physical activity at 7 years was associated with higher hyperactivity and lower peer problems throughout adolescence. Higher moderate‐to‐vigorous (incidence rate ratio [IRR]: 0.95; 95% confidence interval [CI]: 0.93–0.98), light (0.90; 0.85–0.95), and total activity time (0.95; 0.93–0.98) at 7 years were associated with lower, while sedentary time was associated with higher (1.11; 1.07–1.16) psychological distress at 17 years in the adjusted models. Girls who spent more time in light physical activity at 7 years were more likely to have lower hyperactivity levels over adolescence, while higher sedentary time at the same age was associated with higher hyperactivity levels. Higher sedentary time at 7 years was associated with higher depressive symptoms at 14 years (1.06; 1.01–1.10), whereas light physical activity at 7 years (0.91; 0.85–0.97) was associated with lower depressive symptoms at 14 years in the adjusted models.

**Conclusion:**

Higher moderate‐to‐vigorous, light physical activity, and lower sedentary time were associated with lower peer problems, depressive symptoms, and psychological distress throughout adolescence.

**Key Practitioner Message**

**What is currently known?**

- Physical activity and sedentary time are associated with later mental health‐related outcomes among youth
- Most of the previous studies were based on self‐reported assessments and had limited follow‐up duration

**What has been shown?**

- We showed that higher moderate‐to‐vigorous physical activity at 7 years was associated with internalizing and externalizing symptoms at different stages of adolescence. This association was different according to different genders

**What is the significance of this for clinical practice?**

- Interventions should target at increasing physical activity and limiting sedentary time during early ages to protect for later negative mental health‐related outcomes

## Introduction

In 2022, approximately 20% of UK youth between the ages of 8 and 16 years presented a probable mental disorder, considering internalizing and externalizing symptoms (Newlove‐Delgado et al., 2023). Internalizing symptoms refer to emotional and psychological difficulties (e.g., depressive symptoms), while externalizing symptoms include behavioral problems, such as aggressive behavior and hyperactivity (Achenbach, Ivanova, Rescorla, Turner, & Althoff, 2016). It is worth noting that adolescence is a critical transitional stage, with most mental disorders having their onset during this period (Solmi et al., 2022). Therefore, the period between childhood and adolescence represents a crucial interval to identify risk and protective factors so that strategies (e.g., engaging in regular physical activity and limiting sedentary behavior) can be timely implemented to mitigate the development of mental disorders later in life (Patton et al., 2014).

Higher levels of physical activity and reduced sedentary time have been recognized as protective factors for various mental health‐related negative outcomes among adults, including depressive disorders (Huang et al., 2020; Pearce et al., 2022; Schuch et al., 2018; Zou et al., 2024; Zhang et al., 2025). However, the prospective associations of physical activity and sedentary time during childhood with mental health‐related outcomes, such as internalizing and externalizing symptoms, during adolescence remain largely unclear. Specifically, the majority of previous studies using self‐reported movement behaviors indicated mixed findings (Rodriguez‐Ayllon et al., 2019; Zhang, Yang, Wang, Han, & Wu, 2022). Although self‐reported methods are valuable for considering different types of physical activity and sedentary behavior, they have several biases, such as recall and desirability bias. They also tend to overestimate physical activity and underestimate sedentary time (Adamo, Prince, Tricco, Connor‐Gorber, & Tremblay, 2009; Atkin et al., 2012). In addition, most self‐report methods are unable to estimate light physical activity, which is the most frequent form of movement behavior in the waking period of the 24‐hr diurnal cycle (Cooper et al., 2015).

The few studies investigating the association of device‐based physical activity and sedentary time with depressive symptoms and psychological distress during childhood and adolescence also presented mixed findings (Ahn, Sera, Cummins, & Flouri, 2018; Hamer, Patalay, Bell, & Batty, 2020; Jiang et al., 2023; Kandola, Lewis, Osborn, Stubbs, & Hayes, 2020; Kracht, Pochana, & Staiano, 2023; Toseeb et al., 2014). The use of device‐based methods to estimate physical activity and sedentary time may also be subject to different biases due to the different decisions made during data processing, such as epoch length, minimum wear time and device placement (Migueles et al., 2017). Although there are few studies that investigated the associations of physical activity and sedentary time with externalizing problems, such as attention‐deficit hyperactivity disorder, there is also mixed evidence, in which physical activity can be protective or even associated with higher externalizing symptoms (Ahn et al., 2018; Ganjeh et al., 2022; Wiklund, Ekblom, Wang, & Ekblom, 2025). Beyond the mixed findings, studies on how physical activity and sedentary time during childhood could be associated with later mental health‐related outcomes, including depressive symptoms and psychological distress during different stages of adolescence are still warranted.

Identifying how physical activity and sedentary time during early ages are longitudinally associated with later mental health‐related outcomes could help health professionals and school managers foster the development of early preventive strategies. For instance, physical activity shows a moderate tracking from childhood to adolescence (Kwon et al., 2012; Ramos‐Munell et al., 2024; Smith, Gardner, Aggio, & Hamer, 2015). This suggests that early interventions, particularly involving moderate‐to‐vigorous activities such as sports and exercise practices, could potentially have a long‐term effect. Therefore, we aimed to analyze the association of device‐based physical activity and sedentary time during childhood (i.e., 7 years) with internalizing and externalizing symptoms throughout adolescence (i.e., 11 years, 14 years, and 17 years), as well as specifically depressive symptoms (i.e., 14 years) and psychological distress (i.e., 17 years).

## Methods

### Sample

Data from the Millennium Cohort Study (MCS) were used. MCS is a cohort aiming to collect a broad multidisciplinary range of information, including (but not limited to) sociodemographic, behavioral and health‐related factors throughout the members' lives. MCS included a representative sample of children born between September 1, 2000 and August 31, 2001 in England and Wales, and from November 24, 2000 to January 11, 2002 in Scotland and Northern Ireland, who were alive, living in the United Kingdom, aged 9 months at the time of the first wave, and eligible to receive child benefit. More details about the MCS design were previously published (Connelly & Platt, 2014). To ensure representativity, there was an oversampling of children with lower socioeconomic backgrounds and from ethnic minority areas. We included data from the 2008, 2012, 2015, and 2018 waves, when children were 7, 11, 14, and 17 years old, respectively. The initial sample included data from 18,552 families, comprising 18,827 participants. A total of 14,043 children participated in the 7‐year wave, 13,219 accepted using accelerometers, and 12,625 were included in the accelerometer study. The accelerometer was successfully returned by 10,034 children and 8939 had accelerometer data and 6675 presented at least two valid days. Additionally, 320 participants presented missing data on baseline confounders. From the base sample of 6355 children, 6009 presented complete data at 11 years, 5381 presented complete data at 14 years and 4586 presented complete data at 17 years. Ethics approval for all procedures was obtained from the Northern and Yorkshire Multi‐Centre Research Ethics Committee of the NHS. Informed consent was secured from all participating families.

### Device‐based physical activity and sedentary behavior

Device‐based physical activity and sedentary behavior were assessed using the ActiGraph GT1M accelerometer (ActiGraph, Pensacola, Florida, USA). Child participants in the 7‐year wave were invited to use accelerometers between May 2008 and August 2009. Those who agreed to participate were instructed to use the devices on top of the right hip. The participants were also instructed to wear the accelerometers for seven consecutive days during waking time, except for bathing and aquatic activities.

The accelerometers were set to record epochs of 15 s. Periods of consecutive zero counts of 20 min or more were considered as non‐wear time (Migueles et al., 2017). A valid day was defined as those with at least 10 hr of wear time during the waking day (Rich et al., 2014). Participants with at least two valid days of data were included in the analysis. As cutoff points for physical activity intensities and sedentary behavior, we adopted <100 counts/min for sedentary behavior, 100–2240 counts/min for light physical activity, and 2241–11,714 counts/min for moderate‐to‐vigorous physical activity (Pulsford et al., 2011). Extreme values of counts/min were considered as outliers and possible non‐reliable values due to measurement errors (Rich et al., 2014). To improve the interpretability of the physical activity and sedentary time indicators in the main analysis, we divided sedentary time and light physical activity for 60 min (i.e., exposure meaning increases/reductions in units of 60 min), moderate‐to‐vigorous for 15 min (i.e., exposure meaning increases/reductions in units of 15 min), and total activity for 100 counts/min (i.e., exposure meaning increases/reductions in units of 100 counts/min) (Kandola et al., 2020).

### Mental health‐related outcomes

Mental health‐related outcomes included internalizing (i.e., emotional and peer problems) and externalizing symptoms (i.e., hyperactivity and conduct problems), which were assessed at 11, 14, and 17 years and answered by the main caregiver. Depressive symptoms were included at 14 years, and psychological distress symptoms were included at 17 years. Both depressive symptoms and psychological distress scales were self‐reported by the participants.

The Strengths and Difficulties Questionnaire (SDQ) was used to estimate internalizing and externalizing symptoms (Goodman, 1997). The SDQ includes five subscales regarding different areas of difficulties (emotional symptoms, conduct problems, hyperactivity, peer problems, and prosocial behavior), comprising five 3‐point Likert scale items each and a score ranging between 0 and 10. Higher scores in each scale represent higher difficulties. Considering a previous manuscript, we did not consider prosocial behavior (Ahn et al., 2018). In addition, having in mind that our focus was to estimate the association in the different subscales as they represent different symptoms, we did not include a total score. Depressive symptoms were assessed using the Mood and Feelings Questionnaire (Angold, Costello, Messer, & Pickles, 1995). This questionnaire includes 13 items comprising experiences during the prior 2 weeks in which the participant answers how true each one is on a 3‐point Likert scale response. The final score ranges between 0 and 26, with higher scores reflecting higher presence of depressive symptoms. Psychological distress was assessed using the 6‐item Kessler psychological distress scale (Kessler et al., 2002). The scale includes different feelings (i.e., nervousness, hopelessness, lack of energy, depressive mood, restlessness, and worthlessness), and the participant answers the frequency in which the participant experiences each item in a 5‐item Likert scale. Total scores ranged from 0 to 24, with higher scores representing higher psychological distress.

### Potential confounders

Potential confounders were selected based on previous literature (Ahn et al., 2018; Kandola et al., 2020). We included age at the first wave (i.e., 2008), mean accelerometer wear time, season of accelerometer use, ethnicity, maternal education, maternal psychological distress, internalizing and externalizing symptoms at 7 years. Age was used continuously. Total accelerometer wear time was derived from the mean time of the valid accelerometer days. The season of accelerometer use was computed based on the first valid day of accelerometer use. Ethnicity was self‐reported by the main caregiver, and classified as White, Mixed, Indian, Pakistani, or Bangladeshi, Black or Black British, or other. Maternal education was assessed considering the highest academic achievement. Maternal psychological distress was assessed using the Kessler psychological distress scale (Kessler et al., 2002). Internalizing and externalizing symptoms were estimated using the main caregiver report of the SDQ at 7 years as described in the previous section.

### Statistical analysis

Values of mean and 95% confidence interval (95% CI) for continuous variables as well as frequency distribution for categorical variables were used to describe the characteristics of the sample. We have imputed missing values for the potential confounders by using the Hot Deck method according to gender (Age at the first wave (*n* = 17), ethnicity (*n* = 17), maternal education (*n* = 546), maternal psychological distress (*n* = 395), internalizing (*n* = 121), and externalizing symptoms (*n* = 125) at 7 years) (Andridge & Little, 2010). Characteristics of the sample considering the participants included for the outcomes of each wave of follow‐up were also estimated. Possible gender differences were assessed using the 95% CI as well as the chi‐square test. The linearity was tested using restricted cubic splines, defining three knots, and comparing these models with a linear term, using the likelihood‐ratio test. There were no differences between the model with continuous and restricted cubic spline for each indicator. Therefore, continuous exposures were used in the analysis. Considering the overdispersion of outcomes, negative binomial regression models were used, estimating the incidence rate ratio (IRR) to estimate the prospective association of physical activity and sedentary time during childhood with internalizing and externalizing symptoms throughout adolescence as well as with depressive symptoms during mid‐adolescence and psychological distress during late adolescence. Two sets of models were created. First, unadjusted models were created, including one model for each behavior. Second, separate models for each behavior were created, adjusting for covariates (i.e., age, mean accelerometer wear time, season of accelerometer use, ethnicity, maternal education, maternal psychological distress, internalizing and externalizing symptoms at 7 years). All the analyses accounted for appropriate sampling weights and were conducted using the software Stata 18. The codes for the main analysis are provided in Appendix S1.

## Results

The characteristics of the sample at 7 years are presented in Table 1. Boys showed slightly higher light physical activity and lower sedentary time, while they also had higher moderate‐to‐vigorous physical activity and total movement. Internalizing symptoms were similar among boys and girls, but boys consistently showed higher levels of externalizing symptoms, including hyperactivity and conduct problems. There were only minor differences for the included sample in each analysis (Table S1).

**Table 1:** Baseline characteristics of the sample

Variables	Category or measure	Whole sample (*n* = 6355)	Girls (*n* = 3262)	Boys (*n* = 3093)
Mother's psychological distress	Score	2.90 (2.77–3.03)	2.85 (2.67–3.04)	3.00 (1.84–3.16)
Mother's education	Less than high school	54.8%	54.1%	55.2%
	High school or more	45.2%	45.9%	44.8%
Ethnicity	White	85.2%	85.1%	86.4%
	Mixed	3.2%	3.4%	2.7%
	Indian	2.0%	1.8%	2.3%
	Pakistani or Bangladeshi	5.1%	5.2%	4.2%
	Black or Black British	3.2%	3.4%	2.6%
	Other	1.4%	1.1%	1.7%
Light physical activity	min/day	281.2 (279.8–282.6)	278.4 (276.6–280.3)	282.9 (280.8–285.1)
Moderate‐to‐vigorous physical activity	min/day	63.28 (62.48–64.08)	56.24 (55.37–57.11)	69.73 (68.53–70.94)
Sedentary behavior	min/day	390.9 (388.5–393.3)	396.17 (392.85–399.49)	384.9 (381.8–387.9)
Total movement	100 counts/min	6.10 (6.04–6.15)	5.74 (5.67–5.81)	6.43 (6.35–6.51)
Mean wear time	min/day	735.4 (733.1–737.6)	730.8 (727.8–733.9)	737.5 (734.7–740.4)
Season of assessment	Spring	11.5%	12.1%	11.1%
	Summer	45.3%	46.3%	44.5%
	Autumn	33.7%	32.3%	35.0%
	Winter	9.5%	9.3%	9.4%
*Internalizing and externalizing symptoms*				
Emotion problems at 7 years	Score	1.50 (1.44–1.56)	1.53 (1.46–1.60)	1.44 (1.36–1.53)
Peer problem at 7 years	Score	1.19 (1.13–1.24)	1.10 (1.02–1.17)	1.25 (1.17–1.33)
Hyperactivity at 7 years	Score	3.29 (3.21–3.37)	2.84 (2.74–2.95)	3.69 (3.57–3.80)
Conduct problems at 7 years	Score	1.37 (1.32–1.43)	1.21 (1.15–1.28)	1.51 (1.43–1.58)

Mean and 95% confidence intervals were used for continuous variables, and relative frequencies were used for categorical variables.

The adjusted models of the association of physical activity and sedentary time during childhood with internalizing and externalizing symptoms over adolescence are shown in Table 2. Among boys, moderate‐to‐vigorous and total physical activity were associated with lower peer problems at all time points, while sedentary time was associated with higher peer problems at 11 years and 17 years. There were no significant associations between physical activity, sedentary time, and internalizing symptoms among girls. Higher moderate‐to‐vigorous, light, and total physical activity at 7 years were linked to higher hyperactivity among both genders at 11 years and among boys at 14 years, while light physical activity and total physical activity at 7 years were also related to higher hyperactivity among girls at 14 years. Only light physical activity showed a positive association with higher hyperactivity and conduct problems, among girls, at 17 years.

**Table 2:** Models of the association of physical activity intensities and sedentary time with internalizing and externalizing symptoms during different stages of adolescence

	11 years		14 years		17 years	
	Boys IRR (95% CI)	Girls IRR (95% CI)	Boys IRR (95% CI)	Girls IRR (95% CI)	Boys IRR (95% CI)	Girls IRR (95% CI)
*Emotion problems*						
MVPA, per 15 min	0.99 (0.95–1.02)	1.01 (0.97–1.05)	1.02 (0.98–1.06)	1.00 (0.95–1.05)	0.97 (0.93–1.01)	1.00 (0.96–1.05)
LPA, per 60 min	1.00 (0.93–1.08)	1.03 (0.96–1.12)	1.08 (0.99–1.18)	0.97 (0.90–1.05)	0.97 (0.88–1.07)	1.08 (0.99–1.17)
Sedentary behavior, per 60 min	1.01 (0.95–1.08)	0.97 (0.91–1.03)	0.94 (0.87–1.01)	1.02 (0.96–1.08)	1.05 (0.97–1.13)	0.95 (0.89–1.01)
Total activity, per 100 counts/min	0.99 (0.95–1.02)	1.02 (0.98–1.05)	1.03 (0.98–1.07)	1.00 (0.96–1.05)	0.97 (0.93–1.01)	1.01 (0.97–1.06)
*Peer problem*						
MVPA, per 15 min	**0.95 (0.92–0.98)**	1.03 (0.97–1.09)	**0.95 (0.92–0.98)**	1.02 (0.97–1.06)	**0.94 (0.91–0.97)**	1.03 (0.98–1.08)
LPA, per 60 min	0.93 (0.85–1.00)	0.92 (0.84–1.00)	1.04 (0.96–1.13)	0.94 (0.87–1.01)	0.96 (0.88–1.04)	1.01 (0.94–1.09)
Sedentary behavior, per 60 min	**1.10 (1.03–1.17)**	1.03 (0.97–1.10)	1.02 (0.96–1.08)	1.03 (0.97–1.10)	**1.08 (1.02–1.15)**	0.97 (0.92–1.03)
Total activity, per 100 counts/min	**0.95 (0.92–0.99)**	1.01 (0.96–1.07)	**0.96 (0.93–0.99)**	1.01 (0.97–1.05)	**0.94 (0.92–0.97)**	1.02 (0.98–1.07)
*Hyperactivity*						
MVPA, per 15 min	**1.03 (1.01–1.05)**	1.02 (1.00–1.04)	**1.05 (1.02–1.07)**	1.02 (0.99–1.05)	1.02 (0.99–1.05)	1.03 (0.99–1.08)
LPA, per 60 min	**1.06 (1.01–1.10)**	**1.11 (1.05–1.17)**	**1.10 (1.03–1.16)**	**1.08 (1.02–1.14)**	1.04 (0.97–1.12)	**1.08 (1.02–1.16)**
Sedentary behavior, per 60 min	**0.94 (0.91–0.97)**	**0.93 (0.89–0.96)**	**0.91 (0.87–0.95)**	**0.94 (0.90–0.98)**	0.96 (0.91–1.01)	**0.93 (0.88–0.98)**
Total activity, per 100 counts/min	**1.03 (1.02–1.05)**	**1.02 (1.01–1.05)**	**1.05 (1.03–1.07)**	1.03 (1.00–1.05)	1.02 (0.99–1.05)	1.03 (0.99–1.07)
*Conduct problems*						
MVPA, per 15 min	1.03 (1.00–1.06)	1.01 (0.97–1.04)	1.03 (0.99–1.06)	1.01 (0.97–1.06)	1.03 (0.99–1.07)	1.03 (0.96–1.11)
LPA, per 60 min	0.98 (0.90–1.06)	1.01 (0.94–1.10)	1.04 (0.95–1.14)	1.03 (0.96–1.11)	1.09 (0.97–1.22)	**1.11 (1.01–1.22)**
Sedentary behavior, per 60 min	0.98 (0.92–1.05)	0.99 (0.93–1.05)	0.95 (0.89–1.02)	0.97 (0.91–1.03)	0.93 (0.85–1.01)	**0.92 (0.84–0.99)**
Total activity, per 100 counts/min	1.03 (1.00–1.07)	1.01 (0.97–1.04)	1.03 (0.99–1.07)	1.01 (0.98–1.05)	1.04 (1.00–1.07)	1.03 (0.97–1.10)

Adjusted for age at the first wave (i.e., 2008), mean accelerometer wear time, season of accelerometer use, ethnicity, maternal education, maternal psychological distress, internalizing and externalizing symptoms at 7 years. IRR, incidence rate ratio. CI, confidence interval. Values in bold represent *p* < 0.05.

Table 3 shows the adjusted associations of physical activity and sedentary time with depressive symptoms at 14 years and psychological distress at 17 years. Higher sedentary time at 7 years was associated with depressive symptoms among both genders at 14 years, while light physical activity predicted lower depressive symptoms among girls. For boys, higher moderate‐to‐vigorous, light, and total physical activity at 7 years was linked to lower psychological distress at 17 years and higher sedentary time was related to higher psychological distress at 17 years. Among girls, no significant associations were observed between physical activity and sedentary time and psychological distress.

**Table 3:** Models of the association of physical activity intensities and sedentary time at 7 years with depressive symptoms at 14 years and psychological distress at 17 years

	Depressive symptoms at 14 years		Psychological distress at 17 years	
	Boys IRR (95% CI)	Girls IRR (95% CI)	Boys IRR (95% CI)	Girls IRR (95% CI)
MVPA, per 15 min	0.97 (0.94–1.01)	1.01 (0.96–1.05)	**0.95 (0.93–0.98)**	1.02 (0.99–1.04)
LPA, per 60 min	0.93 (0.85–1.02)	**0.91 (0.85–0.97)**	**0.90 (0.85–0.95)**	0.96 (0.92–1.01)
Sedentary behavior, per 60 min	**1.07 (1.01–1.15)**	**1.06 (1.01–1.12)**	**1.11 (1.07–1.16)**	1.01 (0.98–1.05)
Total activity, per 100 counts/min	0.98 (0.94–1.01)	0.99 (0.96–1.04)	**0.95 (0.93–0.98)**	1.01 (0.99–1.03)

Adjusted for age at the first wave (i.e., 2008), mean accelerometer wear time, season of accelerometer use, ethnicity, maternal education, maternal psychological distress, internalizing and externalizing symptoms at 7 years. IRR, incidence rate ratio. CI, confidence interval. Values in bold represent *p* < 0.05.

Unadjusted models for the associations of physical activity and sedentary time with internalizing and externalizing symptoms (Table S2), and depressive symptoms and psychological distress (Table S3) over adolescence showed slightly higher coefficients than adjusted analysis, with minor differences. The major differences between unadjusted and adjusted models were in the analysis of hyperactivity, in which most of the associations found in the unadjusted models were lost or reduced in the adjusted models.

## Discussion

The present study aimed to investigate the association between different intensities of physical activity and sedentary time during childhood and indicators of internalizing and externalizing problems at different stages of adolescence, as well as depressive symptoms in mid‐adolescence and psychological distress in late adolescence. The main findings were that higher moderate‐to‐vigorous physical activity at 7 years was associated with fewer peer problems and greater hyperactivity symptoms throughout adolescence among boys. Among girls, light physical activity was associated with higher hyperactivity symptoms at 14 years, while higher sedentary time was associated with lower symptoms. Additionally, physical activity at different intensities and sedentary time at 7 years were linked to lower psychological distress at 17 years in boys, while higher sedentary time at 7 years was associated with higher depressive symptoms in both genders and higher light physical activity at 7 years was associated with lower depressive symptoms at 14 years among girls.

The present findings align with previous research, which has indicated that higher levels of physical activity and lower sedentary time are associated with increased hyperactivity symptoms and decreased peer problems (Ahn et al., 2018). However, this study advances this understanding by demonstrating that these associations persist into mid‐adolescence for hyperactivity and into late adolescence for peer problems. These findings may indicate a long‐term association of physical activity with mental health‐related outcomes, showing an association between childhood physical activity and reduced psychological distress at 17 years. There are mixed findings on the prospective association between device‐based physical activity and sedentary time and depressive symptoms or psychological distress, which may be derived from methodological differences, especially in device‐based methods. Some studies report associations between physical activity and later depressive symptoms and psychological distress (Jiang et al., 2023; Kandola et al., 2020; Kracht et al., 2023), while others find no associations (Hamer et al., 2020; Toseeb et al., 2014). Differences in study results include sample size, which can affect analytical power, the use of categorical exposure measures, as well as potential differences in the distribution of physical activity domains, that may vary according to the population but are not captured by device‐based methods.

We also observed that the association between device‐based physical activity during childhood and internalizing and externalizing symptoms in early adolescence diminished through mid‐ and late adolescence. This finding is consistent with previous research indicating that the association between moderate‐to‐vigorous physical activity and depressive symptoms at 18 years is stronger considering device‐based assessments during early adolescence compared with middle‐ and late adolescence, possibly due to a decrease in physical activity during adolescence as well as possible changes in the domains of physical activity practiced, such as increasing transport and reducing non‐organized leisure‐time physical activity (Cooper et al., 2015; Hamer et al., 2020; Kandola et al., 2020; Kemp, Parrish, Batterham, & Cliff, 2020). Gender differences were observed in the present study, with more associations among boys, particularly for moderate‐to‐vigorous physical activity, while girls showed associations between light physical activity and/or sedentary behavior with depressive symptoms at 14 years, psychological distress at 17 years, peer problems at 11 years, and hyperactivity at 11 years and 14 years. These findings might be due to lower levels of moderate‐to‐vigorous physical activity among girls (Kretschmer et al., 2023). Therefore, these findings highlight the importance of device‐based methods to explore the association between movement behaviors during childhood and later mental health‐related outcomes among girls.

Physical activity can be an engaging activity that promotes social interaction with other participants and consequently, increases social integration (Doré et al., 2020; Sabiston et al., 2016). Therefore, part of the association between moderate‐to‐vigorous physical activity and reduced peer problems may be because of a higher social integration influenced by physical activity practices. The positive association between physical activity and hyperactivity also supports findings from previous research (Ahn et al., 2018). Although models adjusted for baseline levels of internalizing and externalizing symptoms, it is possible that the hyperactivity symptoms originated before the baseline, which may represent a residual confounding.

Physical activity may be associated with depressive symptoms and psychological distress through multiple mechanisms, such as reducing inflammation, elevating dopamine release, promoting alteration in neuroplasticity, as well as through psychosocial mechanisms such as stimulating social connectedness and promoting self‐esteem (Agbaje, 2023; Doré et al., 2020; Hou et al., 2024; Kandola, Ashdown‐Franks, Hendrikse, Sabiston, & Stubbs, 2019; Valkenborghs et al., 2019). Although most of the evidence is focused on exercise or moderate‐to‐vigorous physical activity, light physical activity is also hypothesized to present similar mechanisms. Similarly, sedentary time may also be associated with similar mechanisms linking physical activity and depressive symptoms, especially during behaviors involving mentally passive activities, such as watching TV, in which there is a minimal cognitive demand (Hallgren, Dunstan, & Owen, 2020).

Our findings highlight the need for strategies aiming at increasing physical activity and limiting sedentary time during early ages as they are associated with mental health‐related outcomes throughout adolescence. Furthermore, physical activity and sedentary behavior have a moderate level of tracking through childhood and into adulthood (Husøy et al., 2024; Kwon et al., 2012; Ramos‐Munell et al., 2024), suggesting that early childhood interventions can also influence levels of physical activity and sedentary behavior during adolescence and adulthood. Interventions should target groups with lower physical activity levels and higher social vulnerability, such as girls, children from lower socioeconomic backgrounds, and ethnic minorities (Sport England, 2023; Werneck, Barboza, Silva, & Araujo, 2022).

The present study included a large sample of device‐based physical activity and sedentary time data during childhood and investigated their association with mental health‐related outcomes throughout adolescence. Although this was a subsample from the Millennium Cohort Study members, there were no major sociodemographic differences compared with the whole sample (Griffiths et al., 2013). This could translate into a considerable generalizability power when appropriate post‐stratification weights are applied. However, findings should also be interpreted in light of potential limitations. First, although the analysis was adjusted for internalizing and externalizing symptoms during baseline (i.e., 7 years), data were not available on depressive symptoms or psychological distress during the baseline, which may be associated with a residual bias in the analysis of depressive symptoms at 14 years and psychological distress at 17 years. Second, internalizing and externalizing symptoms were reported by a caregiver, which may differ from self‐report, as caregivers tend to underestimate the symptoms (Booth, Moreno‐Agostino, & Fitzsimons, 2023). Third, accelerometers placed at the hip may not capture certain activities, such as resistance training and cycling, and are not waterproof, thus not used in aquatic activities like swimming. Additionally, using accelerometers can cause a reactivity effect, where participants increase their physical activity levels due to wearing the devices. Accelerometers also only capture levels of physical activity and sedentary behavior and not domains; it is possible that differing associations would be observed between levels of physical activity and sedentary behavior spent in varying domains of physical activity and sedentary behavior with mental health outcomes. The decisions made during data processing, such as minimum wear time, epoch and cutoff points may also be associated with substantial variance in the final outputs of time in different movement behaviors (Migueles et al., 2017). Finally, including only two valid days could introduce a bias in representing habitual movement behaviors, although previous studies have used even one valid day, aiming to reduce sample loss (Cooper et al., 2015; Kwon et al., 2012) and others demonstrated a small difference between using 2 or 3 valid days (Janz, Witt, & Mahoney, 1995; Vanhelst, Fardy, Duhamel, & Béghin, 2014). Thus, future research should aim to explore such associations. Fourth, although the number of twins (*n* = 77) and triplets (*n* = 5) was reduced, the nesting of the data may have introduced a slight bias.

## Conclusion

Different physical activity intensities and sedentary time during childhood were associated with mental health‐related outcomes throughout adolescence. Although the associations reduced over time, higher levels of moderate‐to‐vigorous physical activity at 7 years were still associated with lower peer problems and psychological distress at 17 years. Light physical activity also presented an association with lower psychological distress at 17 years for both genders. Future research should explore how physical activity and sedentary time during childhood are associated with other mental health‐related outcomes throughout adolescence (e.g., anxiety and depressive symptoms) as well as explore how trajectories since childhood are associated with these outcomes.

## Conflict of interest statement

B.S. is on the Editorial Board of Ageing Research Reviews, Mental Health and Physical Activity, The Journal of Evidence Based Medicine, and The Brazilian Journal of Psychiatry. B.S. has received honorarium from a co‐edited book on exercise and mental illness (Elsevier), and unrelated advisory work from ASICS, in addition to honorarium and stock options at FitXR LTD. M.S. has received honoraria, has been a consultant for AbbVie, Angelini, Bausch Health, Boehringer Ingelheim, Pharmascience, Lundbeck, Otsuka, Teva. The remaining authors have declared that they have no competing or potential conflicts of interest.

## Funding information

A.O.W. is supported by the São Paulo Research Foundation (FAPESP) with a research internship abroad scholarship (FAPESP process: 2023/00790–3). B.S. holds an NIHR Advanced Fellowship. B.S. is part funded by the NIHR Maudsley Biomedical Research Centre at South London and Maudsley NHS Foundation Trust and King's College London. L.Z. is supported by the Shenzhen Educational Research Funding (zdzb2014), the Shenzhen Science and Technology Innovation Commission (202307313000096), the Social Science Foundation from the China's Ministry of Education (23YJA880093), the Post‐Doctoral Fellowship (2022M711174), and the National Center for Mental Health (Z014). This paper presents independent research. The views expressed in this publication are those of the authors and not necessarily those of the acknowledged institutions. MS is supported by Clinical Research Chair in Evidence‐Based Mental Health, from the Faculty of Medicine, University of Ottawa, Canada.

## Ethical considerations

Informed consent was obtained by both the participants and the main caregiver. All the waves from the Millennium Cohort Study were approved by an appropriate ethics committee. The 7‐year wave was approved by the Yorkshire MREC on 5 February 2008 (process: 07/MRE03/32), the 11‐year wave was approved by the Yorkshire and The Humber – Leeds East Ethics Committee on the 13th December 2011 (process: 11/YH/0203), the 14‐year wave was approved by the London – Central MREC on the April 11, 2014 (process: 13/LO/1786). The 17‐year wave was approved by the National Research Ethics Service (NRES) Research Ethics Committee (REC) North East – York on the October 18, 2017 (process: 17/NE/0341).

## Supporting information


**Table S1.** Characteristics of the sample for the analysis of outcomes in each follow‐up wave.
**Table S2.** Unadjusted models of the association of physical activity intensities and sedentary time with internalizing and externalizing symptoms during different stages of adolescence.
**Table S3.** Unadjusted models of the association of physical activity intensities and sedentary time at 7 years with depressive symptoms at 14 years and psychological distress at 17 years.
**Appendix S1.** Stata codes for the analyses.

## Data Availability

Data for the Millennium Cohort Study used in this paper are available from the UK Data Service. https://www.ukdataservice.ac.uk/.
