# Supplementary material for: Prospective association of device‐based physical activity and sedentary time during childhood with mental health outcomes during adolescence
Source: Child Adolesc Ment Health. 2025 Dec 2;31(2):116–24. doi: 10.1111/camh.70048 (PMC13116047; doi:10.1111/camh.70048)
Supplement: Supplementary file 1 — Table S1. Characteristics of the sample for the analysis of outcomes in each follow‐up wave. Table S2. Unadjusted models of the association of physical activity intensities and sedentary time with internalizing and externalizing symptoms during different stages of adolescence. Table S3. Unadjusted models of the association of physical activity intensities and sedentary time at 7 years with depressive symptoms at 14 years and psychological distress at 17 years. Appendix S1. Stata codes for the analyses. [file CAMH-31-116-s001.docx]

**Prospective association of device-based physical activity and sedentary time during childhood with mental health outcomes during adolescence**

**Supporting Information**

**Table S1.** Characteristics of the sample for the analysis of outcomes in each follow-up wave

| Variable | Category or measure | Valid data at 11y  (n=6,009) | Valid data at 14y  (n=5,381) | Valid data at 17y  (n=4,586) |
| --- | --- | --- | --- | --- |
| Gender | Girl | 49.6% | 50.2% | 51.4% |
| Mother’s psychological distress | Score | 2.92 (2.79-3.04) | 2.87 (2.74-3.00) | 2.85 (2.71-2.99) |
| Mother’s education | Less than high school | 54.3% | 55.0% | 56.3% |
|  | High school or more | 45.8% | 45.0% | 43.7% |
| Ethnicity | White | 86.3% | 85.2% | 85.2% |
|  | Mixed | 3.1% | 3.1% | 3.0% |
|  | Indian | 1.7% | 2.2% | 2.2% |
|  | Pakistani or Bangladeshi | 4.8% | 5.0% | 5.2% |
|  | Black or Black British | 2.7% | 3.0% | 3.0% |
|  | Other | 1.4% | 1.5% | 1.3% |
| Light Physical activity | min/d | 280.8 (279.4-282.2) | 280.7 (279.3-282.2) | 280.4 (278.9-281.9) |
| Moderate-to-vigorous Physical activity | min/d | 62.99 (62.18-63.80) | 62.62 (61.82-63.52) | 62.09 (61.22-62.96) |
| Sedentary behavior | min/d | 391.0 (388.6-393.4) | 391.0 (388.8-393.2) | 392.5 (390.1-394.9) |
| Total movement | 100 counts/min | 6.08 (6.03-6.14) | 6.06 (6.01-6.12) | 6.03 (5.97-6.08) |
| Mean wear time | min/d | 734.8 (732.5-737.0) | 734.3 (732.0-736.6) | 735.0 (732.7-737.3) |
| Season of assessment | Spring | 11.4% | 11.4% | 10.8% |
|  | Summer | 45.7% | 45.6% | 45.8% |
|  | Autumn | 33.5% | 34.2% | 34.5% |
|  | Winter | 9.5% | 8.8% | 8.9% |
| Emotion problems at 7y | score | 1.49 (1.43-1.54) | 1.46 (1.40-1.52) | 1.46 (1.40-1.52) |
| Peer problem at 7y | score | 1.16 (1.11-1.22) | 1.13 (1.07-1.19) | 1.12 (1.06-1.17) |
| Hyperactivity at 7y | score | 3.24 (3.16-3.33) | 3.16 (3.08-3.25) | 3.14 (3.05-3.23) |
| Conduct problems at 7y | score | 1.34 (1.29-1.40) | 1.29 (1.23-1.35) | 1.27 (1.22-1.33) |
|  |  |  |  |  |
| **Outcomes at the follow-up** |  | 11y | 14y | 17y |
| Emotion problems | score | 1.80 (1.73-1.87) | 2.01 (1.92-2.09) | 1.97 (1.89-2.06) |
| Peer problem | score | 1.34 (1.27-1.40) | 1.71 (1.64-1.79) | 1.75 (1.67-1.83) |
| Hyperactivity | score | 3.07 (2.98-3.15) | 2.95 (2.86-3.05) | 2.41 (2.33-2.50) |
| Conduct problems | score | 1.37 (1.31-1.42) | 1.37 (1.31-1.44) | 1.15 (1.09-1.21) |
| Depressive symptoms | score | - | 5.71 (5.50-5.91) | - |
| Psychological distress | score | - | - | 7.36 (7.19-7.52) |

Note. Mean and 95% confidence intervals were used for continuous variables, and relative frequencies were used for categorical variables.

**Table S2**. Unadjusted models of the association of physical activity intensities and sedentary time with internalizing and externalizing symptoms during different stages of adolescence

|  | 11y | |  | 14y | |  | 17y | |
| --- | --- | --- | --- | --- | --- | --- | --- | --- |
|  | Boys  IRR (95%CI) | Girls  IRR (95%CI) |  | Boys  IRR (95%CI) | Girls  IRR (95%CI) |  | Boys  IRR (95%CI) | Girls  IRR (95%CI) |
| Emotion problems |  |  |  |  |  |  |  |  |
|  |  |  |  |  |  |  |  |  |
| MVPA, per 15min | 0.97 (0.94-1.01) | 1.00 (0.96-1.05) |  | 0.99 (0.95-1.03) | 0.99 (0.95-1.04) |  | 0.95 (0.91-1.00) | 1.00 (0.95-1.05) |
| LPA, per 60min | 1.03 (0.95-1.12) | 1.02 (0.95-1.10) |  | 1.09 (0.99-1.19) | 0.99 (0.91-1.07) |  | 0.99 (0.89-1.10) | 1.08 (0.99-1.16) |
| Sedentary behavior, per 60min | 1.00 (0.95-1.05) | 0.97 (0.93-1.02) |  | 0.96 (0.91-1.02) | 1.00 (0.96-1.05) |  | 0.99 (0.93-1.05) | 0.96 (0.92-1.00) |
| Total activity, per 100/min. | 0.98 (0.94-1.02) | 1.02 (0.98-1.05) |  | 1.01 (0.97-1.05) | 1.00 (0.96-1.04) |  | 0.97 (0.93-1.02) | 1.01 (0.97-1.06) |
|  |  |  |  |  |  |  |  |  |
| Peer problem |  |  |  |  |  |  |  |  |
|  |  |  |  |  |  |  |  |  |
| MVPA, per 15min | 0.94 (0.90-0.98) | 1.03 (0.98-1.08) |  | 0.95 (0.92-0.98) | 1.01 (0.97-1.05) |  | 0.94 (0.91-0.97) | 1.03 (0.99-1.08) |
| LPA, per 60min | 0.97 (0.89-1.06) | 0.96 (0.88-1.05) |  | 1.07 (0.99-1.15) | 0.98 (0.90-1.06) |  | 1.00 (0.92-1.08) | 1.04 (0.97-1.12) |
| Sedentary behavior, per 60min | 1.03 (0.98-1.08) | 1.01 (0.96-1.07) |  | 1.00 (0.96-1.06) | 1.04 (0.98-1.11) |  | 1.02 (0.98-1.07) | 0.99 (0.94-1.05) |
| Total activity, per 100/min. | 0.96 (0.92-0.99) | 1.01 (0.97-1.06) |  | 0.96 (0.93-0.99) | 1.00 (0.96-1.03) |  | 0.95 (0.92-0.98) | 1.02 (0.98-1.06) |
|  |  |  |  |  |  |  |  |  |
| Hyperactivity |  |  |  |  |  |  |  |  |
|  |  |  |  |  |  |  |  |  |
| MVPA, per 15min | 1.05 (1.03-1.07) | 1.07 (1.03-1.10) |  | 1.06 (1.04-1.09) | 1.07 (1.03-1.10) |  | 1.04 (1.01-1.06) | 1.06 (1.02-1.11) |
| LPA, per 60min | 1.12 (1.07-1.18) | 1.19 (1.12-1.25) |  | 1.16 (1.10-1.23) | 1.15 (1.08-1.23) |  | 1.11 (1.04-1.18) | 1.17 (1.10-1.24) |
| Sedentary behavior, per 60min | 0.93 (0.90-0.96) | 0.92 (0.88-0.96) |  | 0.93 (0.89-0.97) | 0.91 (0.87-0.95) |  | 0.91 (0.88-0.95) | 0.93 (0.88-0.97) |
| Total activity, per 100/min. | 1.07 (1.05-1.08) | 1.07 (1.04-1.10) |  | 1.07 (1.05-1.09) | 1.07 (1.04-1.11) |  | 1.05 (1.02-1.08) | 1.06 (1.03-1.10) |
|  |  |  |  |  |  |  |  |  |
| Conduct problems |  |  |  |  |  |  |  |  |
|  |  |  |  |  |  |  |  |  |
| MVPA, per 15min | 1.05 (1.02-1.08) | 1.06 (1.01-1.10) |  | 1.04 (1.01-1.08) | 1.06 (1.01-1.10) |  | 1.05 (1.01-1.09) | 1.06 (0.99-1.13) |
| LPA, per 60min | 1.06 (0.97-1.15) | 1.10 (1.01-1.20) |  | 1.12 (1.02-1.22) | 1.10 (1.01-1.19) |  | 1.19 (1.07-1.33) | 1.17 (1.06-1.29) |
| Sedentary behavior, per 60min | 0.96 (0.91-1.00) | 0.95 (0.90-1.00) |  | 0.97 (0.91-1.02) | 0.91 (0.86-0.96) |  | 0.92 (0.86-0.98) | 0.93 (0.87-0.98) |
| Total activity, per 100/min. | 1.06 (1.02-1.09) | 1.06 (1.02-1.10) |  | 1.05 (1.01-1.09) | 1.07 (1.03-1.11) |  | 1.07 (1.03-1.11) | 1.06 (1.00-1.12) |

Note. MVPA, moderate-to-vigorous physical activity. LPA, light physical activity. IRR, incidence rate ratio. CI, confidence interval.

**Table S3.** Unadjusted models of the association of physical activity intensities and sedentary time at 7y with depressive symptoms at 14y and psychological distress at 17y

|  | Depressive symptoms at 14y | |  | Psychological distress at 17y | |
| --- | --- | --- | --- | --- | --- |
|  | Boys  IRR (95%CI) | Girls  IRR (95%CI) |  | Boys  IRR (95%CI) | Girls  IRR (95%CI) |
| MVPA, per 15min | 0.97 (0.94-1.01) | 1.01 (0.97-1.05) |  | 0.96 (0.93-0.98) | 1.02 (1.00-1.04) |
| LPA, per 60min | 0.95 (0.88-1.03) | 0.95 (0.89-1.01) |  | 0.91 (0.87-0.96) | 0.96 (0.92-1.00) |
| Sedentary behavior, per 60min | 1.03 (0.97-1.08) | 1.02 (0.98-1.06) |  | 1.04 (1.01-1.08) | 0.98 (0.95-1.01) |
| Total activity, per 100/min. | 0.98 (0.95-1.02) | 1.01 (0.97-1.04) |  | 0.96 (0.94-0.98) | 1.02 (1.00-1.04) |

Note. Adjusted for age, total accelerometer wear time, season of accelerometer use, ethnicity, maternal education, maternal psychological distress, internalizing and externalizing symptoms at 7y. IRR, incidence rate ratio. CI, confidence interval.

**Appendix S1**. Stata codes for the analyses

*Hot deck imputation

bysort FCCSEX00: hotdeckvar degree_7 DDKESSLER_7 age_7 weight_status_7 internalizing_7 externalizing_7 DDC06E00_7 SEASON_7 if media_validhours_7 < ., suffix("_mm")

***********Unadjusted models***********************************************************************************************

svy: nbreg EPEER_11 c.mvpa_7_15 if gender_7 == 1 , irr

svy: nbreg EPEER_11 c.mvpa_7_15 if gender_7 == 2 , irr

svy: nbreg EPEER_11 c.lpa_7_60 if gender_7 == 1 , irr

svy: nbreg EPEER_11 c.lpa_7_60 if gender_7 == 2 , irr

svy: nbreg EPEER_11 c.sb_7_60 if gender_7 == 1 , irr

svy: nbreg EPEER_11 c.sb_7_60 if gender_7 == 2 , irr

svy: nbreg EPEER_11 c.count_min_17 if gender_7 == 1 , irr

svy: nbreg EPEER_11 c.count_min_17 if gender_7 == 2 , irr

svy: nbreg EEMOTION_11 c.mvpa_7_15 if gender_7 == 1 , irr

svy: nbreg EEMOTION_11 c.mvpa_7_15 if gender_7 == 2 , irr

svy: nbreg EEMOTION_11 c.lpa_7_60 if gender_7 == 1 , irr

svy: nbreg EEMOTION_11 c.lpa_7_60 if gender_7 == 2 , irr

svy: nbreg EEMOTION_11 c.sb_7_60 if gender_7 == 1 , irr

svy: nbreg EEMOTION_11 c.sb_7_60 if gender_7 == 2 , irr

svy: nbreg EEMOTION_11 c.count_min_17 if gender_7 == 1 , irr

svy: nbreg EEMOTION_11 c.count_min_17 if gender_7 == 2 , irr

svy: nbreg FPEER_14 c.mvpa_7_15 if gender_7 == 1 , irr

svy: nbreg FPEER_14 c.mvpa_7_15 if gender_7 == 2 , irr

svy: nbreg FPEER_14 c.lpa_7_60 if gender_7 == 1 , irr

svy: nbreg FPEER_14 c.lpa_7_60 if gender_7 == 2 , irr

svy: nbreg FPEER_14 c.sb_7_60 if gender_7 == 1 , irr

svy: nbreg FPEER_14 c.sb_7_60 if gender_7 == 2 , irr

svy: nbreg FPEER_14 c.count_min_17 if gender_7 == 1 , irr

svy: nbreg FPEER_14 c.count_min_17 if gender_7 == 2 , irr

svy: nbreg FEMOTION_14 c.mvpa_7_15 if gender_7 == 1 , irr

svy: nbreg FEMOTION_14 c.mvpa_7_15 if gender_7 == 2 , irr

svy: nbreg FEMOTION_14 c.lpa_7_60 if gender_7 == 1 , irr

svy: nbreg FEMOTION_14 c.lpa_7_60 if gender_7 == 2 , irr

svy: nbreg FEMOTION_14 c.sb_7_60 if gender_7 == 1 , irr

svy: nbreg FEMOTION_14 c.sb_7_60 if gender_7 == 2 , irr

svy: nbreg FEMOTION_14 c.count_min_17 if gender_7 == 1 , irr

svy: nbreg FEMOTION_14 c.count_min_17 if gender_7 == 2 , irr

svy: nbreg GPEER_17 c.mvpa_7_15 if gender_7 == 1 , irrr

svy: nbreg GPEER_17 c.mvpa_7_15 if gender_7 == 2 , irrr

svy: nbreg GPEER_17 c.lpa_7_60 if gender_7 == 1 , irrr

svy: nbreg GPEER_17 c.lpa_7_60 if gender_7 == 2 , irrr

svy: nbreg GPEER_17 c.sb_7_60 if gender_7 == 1 , irrr

svy: nbreg GPEER_17 c.sb_7_60 if gender_7 == 2 , irrr

svy: nbreg GPEER_17 c.count_min_17 if gender_7 == 1 , irrr

svy: nbreg GPEER_17 c.count_min_17 if gender_7 == 2 , irr

svy: nbreg GEMOTION_17 c.mvpa_7_15 if gender_7 == 1 , irrr

svy: nbreg GEMOTION_17 c.mvpa_7_15 if gender_7 == 2 , irrr

svy: nbreg GEMOTION_17 c.lpa_7_60 if gender_7 == 1 , irrr

svy: nbreg GEMOTION_17 c.lpa_7_60 if gender_7 == 2 , irrr

svy: nbreg GEMOTION_17 c.sb_7_60 if gender_7 == 1 , irrr

svy: nbreg GEMOTION_17 c.sb_7_60 if gender_7 == 2 , irrr

svy: nbreg GEMOTION_17 c.count_min_17 if gender_7 == 1 , irrr

svy: nbreg GEMOTION_17 c.count_min_17 if gender_7 == 2 , irr

**********

svy: nbreg ECONDUCT_11 c.mvpa_7_15 if gender_7 == 1 , irr

svy: nbreg ECONDUCT_11 c.mvpa_7_15 if gender_7 == 2 , irr

svy: nbreg ECONDUCT_11 c.lpa_7_60 if gender_7 == 1 , irr

svy: nbreg ECONDUCT_11 c.lpa_7_60 if gender_7 == 2 , irr

svy: nbreg ECONDUCT_11 c.sb_7_60 if gender_7 == 1 , irr

svy: nbreg ECONDUCT_11 c.sb_7_60 if gender_7 == 2 , irr

svy: nbreg ECONDUCT_11 c.count_min_17 if gender_7 == 1 , irr

svy: nbreg ECONDUCT_11 c.count_min_17 if gender_7 == 2 , irr

svy: nbreg EHYPER_11 c.mvpa_7_15 if gender_7 == 1 , irr

svy: nbreg EHYPER_11 c.mvpa_7_15 if gender_7 == 2 , irr

svy: nbreg EHYPER_11 c.lpa_7_60 if gender_7 == 1 , irr

svy: nbreg EHYPER_11 c.lpa_7_60 if gender_7 == 2 , irr

svy: nbreg EHYPER_11 c.sb_7_60 if gender_7 == 1 , irr

svy: nbreg EHYPER_11 c.sb_7_60 if gender_7 == 2 , irr

svy: nbreg EHYPER_11 c.count_min_17 if gender_7 == 1 , irr

svy: nbreg EHYPER_11 c.count_min_17 if gender_7 == 2 , irr

svy: nbreg FCONDUCT_14 c.mvpa_7_15 if gender_7 == 1 , irr

svy: nbreg FCONDUCT_14 c.mvpa_7_15 if gender_7 == 2 , irr

svy: nbreg FCONDUCT_14 c.lpa_7_60 if gender_7 == 1 , irr

svy: nbreg FCONDUCT_14 c.lpa_7_60 if gender_7 == 2 , irr

svy: nbreg FCONDUCT_14 c.sb_7_60 if gender_7 == 1 , irr

svy: nbreg FCONDUCT_14 c.sb_7_60 if gender_7 == 2 , irr

svy: nbreg FCONDUCT_14 c.count_min_17 if gender_7 == 1 , irr

svy: nbreg FCONDUCT_14 c.count_min_17 if gender_7 == 2 , irr

svy: nbreg FHYPER_14 c.mvpa_7_15 if gender_7 == 1 , irr

svy: nbreg FHYPER_14 c.mvpa_7_15 if gender_7 == 2 , irr

svy: nbreg FHYPER_14 c.lpa_7_60 if gender_7 == 1 , irr

svy: nbreg FHYPER_14 c.lpa_7_60 if gender_7 == 2 , irr

svy: nbreg FHYPER_14 c.sb_7_60 if gender_7 == 1 , irr

svy: nbreg FHYPER_14 c.sb_7_60 if gender_7 == 2 , irr

svy: nbreg FHYPER_14 c.count_min_17 if gender_7 == 1 , irr

svy: nbreg FHYPER_14 c.count_min_17 if gender_7 == 2 , irr

svy: nbreg GCONDUCT_17 c.mvpa_7_15 if gender_7 == 1 , irr

svy: nbreg GCONDUCT_17 c.mvpa_7_15 if gender_7 == 2 , irr

svy: nbreg GCONDUCT_17 c.lpa_7_60 if gender_7 == 1 , irr

svy: nbreg GCONDUCT_17 c.lpa_7_60 if gender_7 == 2 , irr

svy: nbreg GCONDUCT_17 c.sb_7_60 if gender_7 == 1 , irr

svy: nbreg GCONDUCT_17 c.sb_7_60 if gender_7 == 2 , irr

svy: nbreg GCONDUCT_17 c.count_min_17 if gender_7 == 1 , irr

svy: nbreg GCONDUCT_17 c.count_min_17 if gender_7 == 2 , irr

svy: nbreg GHYPER_17 c.mvpa_7_15 if gender_7 == 1 , irr

svy: nbreg GHYPER_17 c.mvpa_7_15 if gender_7 == 2 , irr

svy: nbreg GHYPER_17 c.lpa_7_60 if gender_7 == 1 , irr

svy: nbreg GHYPER_17 c.lpa_7_60 if gender_7 == 2 , irr

svy: nbreg GHYPER_17 c.sb_7_60 if gender_7 == 1 , irr

svy: nbreg GHYPER_17 c.sb_7_60 if gender_7 == 2 , irr

svy: nbreg GHYPER_17 c.count_min_17 if gender_7 == 1 , irr

svy: nbreg GHYPER_17 c.count_min_17 if gender_7 == 2 , irr

svy: nbreg MF_TOT_14 c.mvpa_7_15 if gender_7 == 1 , irr

svy: nbreg MF_TOT_14 c.mvpa_7_15 if gender_7 == 2 , irr

svy: nbreg MF_TOT_14 c.lpa_7_60 if gender_7 == 1 , irr

svy: nbreg MF_TOT_14 c.lpa_7_60 if gender_7 == 2 , irr

svy: nbreg MF_TOT_14 c.sb_7_60 if gender_7 == 1 , irr

svy: nbreg MF_TOT_14 c.sb_7_60 if gender_7 == 2 , irr

svy: nbreg MF_TOT_14 c.count_min_17 if gender_7 == 1 , irr

svy: nbreg MF_TOT_14 c.count_min_17 if gender_7 == 2 , irr

svy: nbreg GDCKESSL_17 c.mvpa_7_15 if gender_7 == 1 , irr

svy: nbreg GDCKESSL_17 c.mvpa_7_15 if gender_7 == 2 , irr

svy: nbreg GDCKESSL_17 c.lpa_7_60 if gender_7 == 1 , irr

svy: nbreg GDCKESSL_17 c.lpa_7_60 if gender_7 == 2 , irr

svy: nbreg GDCKESSL_17 c.sb_7_60 if gender_7 == 1 , irr

svy: nbreg GDCKESSL_17 c.sb_7_60 if gender_7 == 2 , irr

svy: nbreg GDCKESSL_17 c.count_min_17 if gender_7 == 1 , irr

svy: nbreg GDCKESSL_17 c.count_min_17 if gender_7 == 2 , irr

***********Adjusted models***********************************************************************************************

svy: nbreg EPEER_11 c.mvpa_7_15 gender_7 media_validhours_7 degree_7_mm DDKESSLER_7_mm age_7_mm internalizing_7_mm externalizing_7_mm DDC06E00_7_mm SEASON_7_mm if gender_7 == 1 , irr

svy: nbreg EPEER_11 c.mvpa_7_15 gender_7 media_validhours_7 degree_7_mm DDKESSLER_7_mm age_7_mm internalizing_7_mm externalizing_7_mm DDC06E00_7_mm SEASON_7_mm if gender_7 == 2 , irr

svy: nbreg EPEER_11 c.lpa_7_60 gender_7 media_validhours_7 degree_7_mm DDKESSLER_7_mm age_7_mm internalizing_7_mm externalizing_7_mm DDC06E00_7_mm SEASON_7_mm if gender_7 == 1 , irr

svy: nbreg EPEER_11 c.lpa_7_60 gender_7 media_validhours_7 degree_7_mm DDKESSLER_7_mm age_7_mm internalizing_7_mm externalizing_7_mm DDC06E00_7_mm SEASON_7_mm if gender_7 == 2 , irr

svy: nbreg EPEER_11 c.sb_7_60 gender_7 media_validhours_7 degree_7_mm DDKESSLER_7_mm age_7_mm internalizing_7_mm externalizing_7_mm DDC06E00_7_mm SEASON_7_mm if gender_7 == 1 , irr

svy: nbreg EPEER_11 c.sb_7_60 gender_7 media_validhours_7 degree_7_mm DDKESSLER_7_mm age_7_mm internalizing_7_mm externalizing_7_mm DDC06E00_7_mm SEASON_7_mm if gender_7 == 2 , irr

svy: nbreg EPEER_11 c.count_min_17 gender_7 media_validhours_7 degree_7_mm DDKESSLER_7_mm age_7_mm internalizing_7_mm externalizing_7_mm DDC06E00_7_mm SEASON_7_mm if gender_7 == 1 , irr

svy: nbreg EPEER_11 c.count_min_17 gender_7 media_validhours_7 degree_7_mm DDKESSLER_7_mm age_7_mm internalizing_7_mm externalizing_7_mm DDC06E00_7_mm SEASON_7_mm if gender_7 == 2 , irr

svy: nbreg EEMOTION_11 c.mvpa_7_15 gender_7 media_validhours_7 degree_7_mm DDKESSLER_7_mm age_7_mm internalizing_7_mm externalizing_7_mm DDC06E00_7_mm SEASON_7_mm if gender_7 == 1 , irr

svy: nbreg EEMOTION_11 c.mvpa_7_15 gender_7 media_validhours_7 degree_7_mm DDKESSLER_7_mm age_7_mm internalizing_7_mm externalizing_7_mm DDC06E00_7_mm SEASON_7_mm if gender_7 == 2 , irr

svy: nbreg EEMOTION_11 c.lpa_7_60 gender_7 media_validhours_7 degree_7_mm DDKESSLER_7_mm age_7_mm internalizing_7_mm externalizing_7_mm DDC06E00_7_mm SEASON_7_mm if gender_7 == 1 , irr

svy: nbreg EEMOTION_11 c.lpa_7_60 gender_7 media_validhours_7 degree_7_mm DDKESSLER_7_mm age_7_mm internalizing_7_mm externalizing_7_mm DDC06E00_7_mm SEASON_7_mm if gender_7 == 2 , irr

svy: nbreg EEMOTION_11 c.sb_7_60 gender_7 media_validhours_7 degree_7_mm DDKESSLER_7_mm age_7_mm internalizing_7_mm externalizing_7_mm DDC06E00_7_mm SEASON_7_mm if gender_7 == 1 , irr

svy: nbreg EEMOTION_11 c.sb_7_60 gender_7 media_validhours_7 degree_7_mm DDKESSLER_7_mm age_7_mm internalizing_7_mm externalizing_7_mm DDC06E00_7_mm SEASON_7_mm if gender_7 == 2 , irr

svy: nbreg EEMOTION_11 c.count_min_17 gender_7 media_validhours_7 degree_7_mm DDKESSLER_7_mm age_7_mm internalizing_7_mm externalizing_7_mm DDC06E00_7_mm SEASON_7_mm if gender_7 == 1 , irr

svy: nbreg EEMOTION_11 c.count_min_17 gender_7 media_validhours_7 degree_7_mm DDKESSLER_7_mm age_7_mm internalizing_7_mm externalizing_7_mm DDC06E00_7_mm SEASON_7_mm if gender_7 == 2 , irr

svy: nbreg FPEER_14 c.mvpa_7_15 gender_7 media_validhours_7 degree_7_mm DDKESSLER_7_mm age_7_mm internalizing_7_mm externalizing_7_mm DDC06E00_7_mm SEASON_7_mm if gender_7 == 1 , irr

svy: nbreg FPEER_14 c.mvpa_7_15 gender_7 media_validhours_7 degree_7_mm DDKESSLER_7_mm age_7_mm internalizing_7_mm externalizing_7_mm DDC06E00_7_mm SEASON_7_mm if gender_7 == 2 , irr

svy: nbreg FPEER_14 c.lpa_7_60 gender_7 media_validhours_7 degree_7_mm DDKESSLER_7_mm age_7_mm internalizing_7_mm externalizing_7_mm DDC06E00_7_mm SEASON_7_mm if gender_7 == 1 , irr

svy: nbreg FPEER_14 c.lpa_7_60 gender_7 media_validhours_7 degree_7_mm DDKESSLER_7_mm age_7_mm internalizing_7_mm externalizing_7_mm DDC06E00_7_mm SEASON_7_mm if gender_7 == 2 , irr

svy: nbreg FPEER_14 c.sb_7_60 gender_7 media_validhours_7 degree_7_mm DDKESSLER_7_mm age_7_mm internalizing_7_mm externalizing_7_mm DDC06E00_7_mm SEASON_7_mm if gender_7 == 1 , irr

svy: nbreg FPEER_14 c.sb_7_60 gender_7 media_validhours_7 degree_7_mm DDKESSLER_7_mm age_7_mm internalizing_7_mm externalizing_7_mm DDC06E00_7_mm SEASON_7_mm if gender_7 == 2 , irr

svy: nbreg FPEER_14 c.count_min_17 gender_7 media_validhours_7 degree_7_mm DDKESSLER_7_mm age_7_mm internalizing_7_mm externalizing_7_mm DDC06E00_7_mm SEASON_7_mm if gender_7 == 1 , irr

svy: nbreg FPEER_14 c.count_min_17 gender_7 media_validhours_7 degree_7_mm DDKESSLER_7_mm age_7_mm internalizing_7_mm externalizing_7_mm DDC06E00_7_mm SEASON_7_mm if gender_7 == 2 , irr

svy: nbreg FEMOTION_14 c.mvpa_7_15 gender_7 media_validhours_7 degree_7_mm DDKESSLER_7_mm age_7_mm internalizing_7_mm externalizing_7_mm DDC06E00_7_mm SEASON_7_mm if gender_7 == 1 , irr

svy: nbreg FEMOTION_14 c.mvpa_7_15 gender_7 media_validhours_7 degree_7_mm DDKESSLER_7_mm age_7_mm internalizing_7_mm externalizing_7_mm DDC06E00_7_mm SEASON_7_mm if gender_7 == 2 , irr

svy: nbreg FEMOTION_14 c.lpa_7_60 gender_7 media_validhours_7 degree_7_mm DDKESSLER_7_mm age_7_mm internalizing_7_mm externalizing_7_mm DDC06E00_7_mm SEASON_7_mm if gender_7 == 1 , irr

svy: nbreg FEMOTION_14 c.lpa_7_60 gender_7 media_validhours_7 degree_7_mm DDKESSLER_7_mm age_7_mm internalizing_7_mm externalizing_7_mm DDC06E00_7_mm SEASON_7_mm if gender_7 == 2 , irr

svy: nbreg FEMOTION_14 c.sb_7_60 gender_7 media_validhours_7 degree_7_mm DDKESSLER_7_mm age_7_mm internalizing_7_mm externalizing_7_mm DDC06E00_7_mm SEASON_7_mm if gender_7 == 1 , irr

svy: nbreg FEMOTION_14 c.sb_7_60 gender_7 media_validhours_7 degree_7_mm DDKESSLER_7_mm age_7_mm internalizing_7_mm externalizing_7_mm DDC06E00_7_mm SEASON_7_mm if gender_7 == 2 , irr

svy: nbreg FEMOTION_14 c.count_min_17 gender_7 media_validhours_7 degree_7_mm DDKESSLER_7_mm age_7_mm internalizing_7_mm externalizing_7_mm DDC06E00_7_mm SEASON_7_mm if gender_7 == 1 , irr

svy: nbreg FEMOTION_14 c.count_min_17 gender_7 media_validhours_7 degree_7_mm DDKESSLER_7_mm age_7_mm internalizing_7_mm externalizing_7_mm DDC06E00_7_mm SEASON_7_mm if gender_7 == 2 , irr

svy: nbreg GPEER_17 c.mvpa_7_15 gender_7 media_validhours_7 degree_7_mm DDKESSLER_7_mm age_7_mm internalizing_7_mm externalizing_7_mm DDC06E00_7_mm SEASON_7_mm if gender_7 == 1 , irr

svy: nbreg GPEER_17 c.mvpa_7_15 gender_7 media_validhours_7 degree_7_mm DDKESSLER_7_mm age_7_mm internalizing_7_mm externalizing_7_mm DDC06E00_7_mm SEASON_7_mm if gender_7 == 2 , irr

svy: nbreg GPEER_17 c.lpa_7_60 gender_7 media_validhours_7 degree_7_mm DDKESSLER_7_mm age_7_mm internalizing_7_mm externalizing_7_mm DDC06E00_7_mm SEASON_7_mm if gender_7 == 1 , irr

svy: nbreg GPEER_17 c.lpa_7_60 gender_7 media_validhours_7 degree_7_mm DDKESSLER_7_mm age_7_mm internalizing_7_mm externalizing_7_mm DDC06E00_7_mm SEASON_7_mm if gender_7 == 2 , irr

svy: nbreg GPEER_17 c.sb_7_60 gender_7 media_validhours_7 degree_7_mm DDKESSLER_7_mm age_7_mm internalizing_7_mm externalizing_7_mm DDC06E00_7_mm SEASON_7_mm if gender_7 == 1 , irr

svy: nbreg GPEER_17 c.sb_7_60 gender_7 media_validhours_7 degree_7_mm DDKESSLER_7_mm age_7_mm internalizing_7_mm externalizing_7_mm DDC06E00_7_mm SEASON_7_mm if gender_7 == 2 , irr

svy: nbreg GPEER_17 c.count_min_17 if gender_7 == 1 , irr

svy: nbreg GPEER_17 c.count_min_17 if gender_7 == 2 , irr

svy: nbreg GEMOTION_17 c.mvpa_7_15 gender_7 media_validhours_7 degree_7_mm DDKESSLER_7_mm age_7_mm internalizing_7_mm externalizing_7_mm DDC06E00_7_mm SEASON_7_mm if gender_7 == 1 , irr

svy: nbreg GEMOTION_17 c.mvpa_7_15 gender_7 media_validhours_7 degree_7_mm DDKESSLER_7_mm age_7_mm internalizing_7_mm externalizing_7_mm DDC06E00_7_mm SEASON_7_mm if gender_7 == 2 , irr

svy: nbreg GEMOTION_17 c.lpa_7_60 gender_7 media_validhours_7 degree_7_mm DDKESSLER_7_mm age_7_mm internalizing_7_mm externalizing_7_mm DDC06E00_7_mm SEASON_7_mm if gender_7 == 1 , irr

svy: nbreg GEMOTION_17 c.lpa_7_60 gender_7 media_validhours_7 degree_7_mm DDKESSLER_7_mm age_7_mm internalizing_7_mm externalizing_7_mm DDC06E00_7_mm SEASON_7_mm if gender_7 == 2 , irr

svy: nbreg GEMOTION_17 c.sb_7_60 gender_7 media_validhours_7 degree_7_mm DDKESSLER_7_mm age_7_mm internalizing_7_mm externalizing_7_mm DDC06E00_7_mm SEASON_7_mm if gender_7 == 1 , irr

svy: nbreg GEMOTION_17 c.sb_7_60 gender_7 media_validhours_7 degree_7_mm DDKESSLER_7_mm age_7_mm internalizing_7_mm externalizing_7_mm DDC06E00_7_mm SEASON_7_mm if gender_7 == 2 , irr

svy: nbreg GEMOTION_17 c.count_min_17 gender_7 media_validhours_7 degree_7_mm DDKESSLER_7_mm age_7_mm internalizing_7_mm externalizing_7_mm DDC06E00_7_mm SEASON_7_mm if gender_7 == 1 , irr

svy: nbreg GEMOTION_17 c.count_min_17 gender_7 media_validhours_7 degree_7_mm DDKESSLER_7_mm age_7_mm internalizing_7_mm externalizing_7_mm DDC06E00_7_mm SEASON_7_mm if gender_7 == 2 , irr

svy: nbreg ECONDUCT_11 c.mvpa_7_15 gender_7 media_validhours_7 degree_7_mm DDKESSLER_7_mm age_7_mm internalizing_7_mm externalizing_7_mm DDC06E00_7_mm SEASON_7_mm if gender_7 == 1 , irr

svy: nbreg ECONDUCT_11 c.mvpa_7_15 gender_7 media_validhours_7 degree_7_mm DDKESSLER_7_mm age_7_mm internalizing_7_mm externalizing_7_mm DDC06E00_7_mm SEASON_7_mm if gender_7 == 2 , irr

svy: nbreg ECONDUCT_11 c.lpa_7_60 gender_7 media_validhours_7 degree_7_mm DDKESSLER_7_mm age_7_mm internalizing_7_mm externalizing_7_mm DDC06E00_7_mm SEASON_7_mm if gender_7 == 1 , irr

svy: nbreg ECONDUCT_11 c.lpa_7_60 gender_7 media_validhours_7 degree_7_mm DDKESSLER_7_mm age_7_mm internalizing_7_mm externalizing_7_mm DDC06E00_7_mm SEASON_7_mm if gender_7 == 2 , irr

svy: nbreg ECONDUCT_11 c.sb_7_60 gender_7 media_validhours_7 degree_7_mm DDKESSLER_7_mm age_7_mm internalizing_7_mm externalizing_7_mm DDC06E00_7_mm SEASON_7_mm if gender_7 == 1 , irr

svy: nbreg ECONDUCT_11 c.sb_7_60 gender_7 media_validhours_7 degree_7_mm DDKESSLER_7_mm age_7_mm internalizing_7_mm externalizing_7_mm DDC06E00_7_mm SEASON_7_mm if gender_7 == 2 , irr

svy: nbreg ECONDUCT_11 c.count_min_17 gender_7 media_validhours_7 degree_7_mm DDKESSLER_7_mm age_7_mm internalizing_7_mm externalizing_7_mm DDC06E00_7_mm SEASON_7_mm if gender_7 == 1 , irr

svy: nbreg ECONDUCT_11 c.count_min_17 gender_7 media_validhours_7 degree_7_mm DDKESSLER_7_mm age_7_mm internalizing_7_mm externalizing_7_mm DDC06E00_7_mm SEASON_7_mm if gender_7 == 2 , irr

svy: nbreg EHYPER_11 c.mvpa_7_15 gender_7 media_validhours_7 degree_7_mm DDKESSLER_7_mm age_7_mm internalizing_7_mm externalizing_7_mm DDC06E00_7_mm SEASON_7_mm if gender_7 == 1 , irr

svy: nbreg EHYPER_11 c.mvpa_7_15 gender_7 media_validhours_7 degree_7_mm DDKESSLER_7_mm age_7_mm internalizing_7_mm externalizing_7_mm DDC06E00_7_mm SEASON_7_mm if gender_7 == 2 , irr

svy: nbreg EHYPER_11 c.lpa_7_60 gender_7 media_validhours_7 degree_7_mm DDKESSLER_7_mm age_7_mm internalizing_7_mm externalizing_7_mm DDC06E00_7_mm SEASON_7_mm if gender_7 == 1 , irr

svy: nbreg EHYPER_11 c.lpa_7_60 gender_7 media_validhours_7 degree_7_mm DDKESSLER_7_mm age_7_mm internalizing_7_mm externalizing_7_mm DDC06E00_7_mm SEASON_7_mm if gender_7 == 2 , irr

svy: nbreg EHYPER_11 c.sb_7_60 gender_7 media_validhours_7 degree_7_mm DDKESSLER_7_mm age_7_mm internalizing_7_mm externalizing_7_mm DDC06E00_7_mm SEASON_7_mm if gender_7 == 1 , irr

svy: nbreg EHYPER_11 c.sb_7_60 gender_7 media_validhours_7 degree_7_mm DDKESSLER_7_mm age_7_mm internalizing_7_mm externalizing_7_mm DDC06E00_7_mm SEASON_7_mm if gender_7 == 2 , irr

svy: nbreg EHYPER_11 c.count_min_17 gender_7 media_validhours_7 degree_7_mm DDKESSLER_7_mm age_7_mm internalizing_7_mm externalizing_7_mm DDC06E00_7_mm SEASON_7_mm if gender_7 == 1 , irr

svy: nbreg EHYPER_11 c.count_min_17 gender_7 media_validhours_7 degree_7_mm DDKESSLER_7_mm age_7_mm internalizing_7_mm externalizing_7_mm DDC06E00_7_mm SEASON_7_mm if gender_7 == 2 , irr

svy: nbreg FCONDUCT_14 c.mvpa_7_15 gender_7 media_validhours_7 degree_7_mm DDKESSLER_7_mm age_7_mm internalizing_7_mm externalizing_7_mm DDC06E00_7_mm SEASON_7_mm if gender_7 == 1 , irr

svy: nbreg FCONDUCT_14 c.mvpa_7_15 gender_7 media_validhours_7 degree_7_mm DDKESSLER_7_mm age_7_mm internalizing_7_mm externalizing_7_mm DDC06E00_7_mm SEASON_7_mm if gender_7 == 2 , irr

svy: nbreg FCONDUCT_14 c.lpa_7_60 gender_7 media_validhours_7 degree_7_mm DDKESSLER_7_mm age_7_mm internalizing_7_mm externalizing_7_mm DDC06E00_7_mm SEASON_7_mm if gender_7 == 1 , irr

svy: nbreg FCONDUCT_14 c.lpa_7_60 gender_7 media_validhours_7 degree_7_mm DDKESSLER_7_mm age_7_mm internalizing_7_mm externalizing_7_mm DDC06E00_7_mm SEASON_7_mm if gender_7 == 2 , irr

svy: nbreg FCONDUCT_14 c.sb_7_60 gender_7 media_validhours_7 degree_7_mm DDKESSLER_7_mm age_7_mm internalizing_7_mm externalizing_7_mm DDC06E00_7_mm SEASON_7_mm if gender_7 == 1 , irr

svy: nbreg FCONDUCT_14 c.sb_7_60 gender_7 media_validhours_7 degree_7_mm DDKESSLER_7_mm age_7_mm internalizing_7_mm externalizing_7_mm DDC06E00_7_mm SEASON_7_mm if gender_7 == 2 , irr

svy: nbreg FCONDUCT_14 c.count_min_17 gender_7 media_validhours_7 degree_7_mm DDKESSLER_7_mm age_7_mm internalizing_7_mm externalizing_7_mm DDC06E00_7_mm SEASON_7_mm if gender_7 == 1 , irr

svy: nbreg FCONDUCT_14 c.count_min_17 gender_7 media_validhours_7 degree_7_mm DDKESSLER_7_mm age_7_mm internalizing_7_mm externalizing_7_mm DDC06E00_7_mm SEASON_7_mm if gender_7 == 2 , irr

svy: nbreg FHYPER_14 c.mvpa_7_15 gender_7 media_validhours_7 degree_7_mm DDKESSLER_7_mm age_7_mm internalizing_7_mm externalizing_7_mm DDC06E00_7_mm SEASON_7_mm if gender_7 == 1 , irr

svy: nbreg FHYPER_14 c.mvpa_7_15 gender_7 media_validhours_7 degree_7_mm DDKESSLER_7_mm age_7_mm internalizing_7_mm externalizing_7_mm DDC06E00_7_mm SEASON_7_mm if gender_7 == 2 , irr

svy: nbreg FHYPER_14 c.lpa_7_60 gender_7 media_validhours_7 degree_7_mm DDKESSLER_7_mm age_7_mm internalizing_7_mm externalizing_7_mm DDC06E00_7_mm SEASON_7_mm if gender_7 == 1 , irr

svy: nbreg FHYPER_14 c.lpa_7_60 gender_7 media_validhours_7 degree_7_mm DDKESSLER_7_mm age_7_mm internalizing_7_mm externalizing_7_mm DDC06E00_7_mm SEASON_7_mm if gender_7 == 2 , irr

svy: nbreg FHYPER_14 c.sb_7_60 gender_7 media_validhours_7 degree_7_mm DDKESSLER_7_mm age_7_mm internalizing_7_mm externalizing_7_mm DDC06E00_7_mm SEASON_7_mm if gender_7 == 1 , irr

svy: nbreg FHYPER_14 c.sb_7_60 gender_7 media_validhours_7 degree_7_mm DDKESSLER_7_mm age_7_mm internalizing_7_mm externalizing_7_mm DDC06E00_7_mm SEASON_7_mm if gender_7 == 2 , irr

svy: nbreg FHYPER_14 c.count_min_17 gender_7 media_validhours_7 degree_7_mm DDKESSLER_7_mm age_7_mm internalizing_7_mm externalizing_7_mm DDC06E00_7_mm SEASON_7_mm if gender_7 == 1 , irr

svy: nbreg FHYPER_14 c.count_min_17 gender_7 media_validhours_7 degree_7_mm DDKESSLER_7_mm age_7_mm internalizing_7_mm externalizing_7_mm DDC06E00_7_mm SEASON_7_mm if gender_7 == 2 , irr

svy: nbreg GCONDUCT_17 c.mvpa_7_15 gender_7 media_validhours_7 degree_7_mm DDKESSLER_7_mm age_7_mm internalizing_7_mm externalizing_7_mm DDC06E00_7_mm SEASON_7_mm if gender_7 == 1 , irr

svy: nbreg GCONDUCT_17 c.mvpa_7_15 gender_7 media_validhours_7 degree_7_mm DDKESSLER_7_mm age_7_mm internalizing_7_mm externalizing_7_mm DDC06E00_7_mm SEASON_7_mm if gender_7 == 2 , irr

svy: nbreg GCONDUCT_17 c.lpa_7_60 gender_7 media_validhours_7 degree_7_mm DDKESSLER_7_mm age_7_mm internalizing_7_mm externalizing_7_mm DDC06E00_7_mm SEASON_7_mm if gender_7 == 1 , irr

svy: nbreg GCONDUCT_17 c.lpa_7_60 gender_7 media_validhours_7 degree_7_mm DDKESSLER_7_mm age_7_mm internalizing_7_mm externalizing_7_mm DDC06E00_7_mm SEASON_7_mm if gender_7 == 2 , irr

svy: nbreg GCONDUCT_17 c.sb_7_60 gender_7 media_validhours_7 degree_7_mm DDKESSLER_7_mm age_7_mm internalizing_7_mm externalizing_7_mm DDC06E00_7_mm SEASON_7_mm if gender_7 == 1 , irr

svy: nbreg GCONDUCT_17 c.sb_7_60 gender_7 media_validhours_7 degree_7_mm DDKESSLER_7_mm age_7_mm internalizing_7_mm externalizing_7_mm DDC06E00_7_mm SEASON_7_mm if gender_7 == 2 , irr

svy: nbreg GCONDUCT_17 c.count_min_17 gender_7 media_validhours_7 degree_7_mm DDKESSLER_7_mm age_7_mm internalizing_7_mm externalizing_7_mm DDC06E00_7_mm SEASON_7_mm if gender_7 == 1 , irr

svy: nbreg GCONDUCT_17 c.count_min_17 gender_7 media_validhours_7 degree_7_mm DDKESSLER_7_mm age_7_mm internalizing_7_mm externalizing_7_mm DDC06E00_7_mm SEASON_7_mm if gender_7 == 2 , irr

svy: nbreg GHYPER_17 c.mvpa_7_15 gender_7 media_validhours_7 degree_7_mm DDKESSLER_7_mm age_7_mm internalizing_7_mm externalizing_7_mm DDC06E00_7_mm SEASON_7_mm if gender_7 == 1 , irr

svy: nbreg GHYPER_17 c.mvpa_7_15 gender_7 media_validhours_7 degree_7_mm DDKESSLER_7_mm age_7_mm internalizing_7_mm externalizing_7_mm DDC06E00_7_mm SEASON_7_mm if gender_7 == 2 , irr

svy: nbreg GHYPER_17 c.lpa_7_60 gender_7 media_validhours_7 degree_7_mm DDKESSLER_7_mm age_7_mm internalizing_7_mm externalizing_7_mm DDC06E00_7_mm SEASON_7_mm if gender_7 == 1 , irr

svy: nbreg GHYPER_17 c.lpa_7_60 gender_7 media_validhours_7 degree_7_mm DDKESSLER_7_mm age_7_mm internalizing_7_mm externalizing_7_mm DDC06E00_7_mm SEASON_7_mm if gender_7 == 2 , irr

svy: nbreg GHYPER_17 c.sb_7_60 gender_7 media_validhours_7 degree_7_mm DDKESSLER_7_mm age_7_mm internalizing_7_mm externalizing_7_mm DDC06E00_7_mm SEASON_7_mm if gender_7 == 1 , irr

svy: nbreg GHYPER_17 c.sb_7_60 gender_7 media_validhours_7 degree_7_mm DDKESSLER_7_mm age_7_mm internalizing_7_mm externalizing_7_mm DDC06E00_7_mm SEASON_7_mm if gender_7 == 2 , irr

svy: nbreg GHYPER_17 c.count_min_17 gender_7 media_validhours_7 degree_7_mm DDKESSLER_7_mm age_7_mm internalizing_7_mm externalizing_7_mm DDC06E00_7_mm SEASON_7_mm if gender_7 == 1 , irr

svy: nbreg GHYPER_17 c.count_min_17 gender_7 media_validhours_7 degree_7_mm DDKESSLER_7_mm age_7_mm internalizing_7_mm externalizing_7_mm DDC06E00_7_mm SEASON_7_mm if gender_7 == 2 , irr

svy: nbreg MF_TOT_14 c.mvpa_7_15 gender_7 media_validhours_7 degree_7_mm DDKESSLER_7_mm age_7_mm internalizing_7_mm externalizing_7_mm DDC06E00_7_mm SEASON_7_mm if gender_7 == 1 , irr

svy: nbreg MF_TOT_14 c.mvpa_7_15 gender_7 media_validhours_7 degree_7_mm DDKESSLER_7_mm age_7_mm internalizing_7_mm externalizing_7_mm DDC06E00_7_mm SEASON_7_mm if gender_7 == 2 , irr

svy: nbreg MF_TOT_14 c.lpa_7_60 gender_7 media_validhours_7 degree_7_mm DDKESSLER_7_mm age_7_mm internalizing_7_mm externalizing_7_mm DDC06E00_7_mm SEASON_7_mm if gender_7 == 1 , irr

svy: nbreg MF_TOT_14 c.lpa_7_60 gender_7 media_validhours_7 degree_7_mm DDKESSLER_7_mm age_7_mm internalizing_7_mm externalizing_7_mm DDC06E00_7_mm SEASON_7_mm if gender_7 == 2 , irr

svy: nbreg MF_TOT_14 c.sb_7_60 gender_7 media_validhours_7 degree_7_mm DDKESSLER_7_mm age_7_mm internalizing_7_mm externalizing_7_mm DDC06E00_7_mm SEASON_7_mm if gender_7 == 1 , irr

svy: nbreg MF_TOT_14 c.sb_7_60 gender_7 media_validhours_7 degree_7_mm DDKESSLER_7_mm age_7_mm internalizing_7_mm externalizing_7_mm DDC06E00_7_mm SEASON_7_mm if gender_7 == 2 , irr

svy: nbreg MF_TOT_14 c.count_min_17 gender_7 media_validhours_7 degree_7_mm DDKESSLER_7_mm age_7_mm internalizing_7_mm externalizing_7_mm DDC06E00_7_mm SEASON_7_mm if gender_7 == 1 , irr

svy: nbreg MF_TOT_14 c.count_min_17 gender_7 media_validhours_7 degree_7_mm DDKESSLER_7_mm age_7_mm internalizing_7_mm externalizing_7_mm DDC06E00_7_mm SEASON_7_mm if gender_7 == 2 , irr

svy: nbreg GDCKESSL_17 c.mvpa_7_15 gender_7 media_validhours_7 degree_7_mm DDKESSLER_7_mm age_7_mm internalizing_7_mm externalizing_7_mm DDC06E00_7_mm SEASON_7_mm if gender_7 == 1 , irr

svy: nbreg GDCKESSL_17 c.mvpa_7_15 gender_7 media_validhours_7 degree_7_mm DDKESSLER_7_mm age_7_mm internalizing_7_mm externalizing_7_mm DDC06E00_7_mm SEASON_7_mm if gender_7 == 2 , irr

svy: nbreg GDCKESSL_17 c.lpa_7_60 gender_7 media_validhours_7 degree_7_mm DDKESSLER_7_mm age_7_mm internalizing_7_mm externalizing_7_mm DDC06E00_7_mm SEASON_7_mm if gender_7 == 1 , irr

svy: nbreg GDCKESSL_17 c.lpa_7_60 gender_7 media_validhours_7 degree_7_mm DDKESSLER_7_mm age_7_mm internalizing_7_mm externalizing_7_mm DDC06E00_7_mm SEASON_7_mm if gender_7 == 2 , irr

svy: nbreg GDCKESSL_17 c.sb_7_60 gender_7 media_validhours_7 degree_7_mm DDKESSLER_7_mm age_7_mm internalizing_7_mm externalizing_7_mm DDC06E00_7_mm SEASON_7_mm if gender_7 == 1 , irr

svy: nbreg GDCKESSL_17 c.sb_7_60 gender_7 media_validhours_7 degree_7_mm DDKESSLER_7_mm age_7_mm internalizing_7_mm externalizing_7_mm DDC06E00_7_mm SEASON_7_mm if gender_7 == 2 , irr

svy: nbreg GDCKESSL_17 c.count_min_17 gender_7 media_validhours_7 degree_7_mm DDKESSLER_7_mm age_7_mm internalizing_7_mm externalizing_7_mm DDC06E00_7_mm SEASON_7_mm if gender_7 == 1 , irr

svy: nbreg GDCKESSL_17 c.count_min_17 gender_7 media_validhours_7 degree_7_mm DDKESSLER_7_mm age_7_mm internalizing_7_mm externalizing_7_mm DDC06E00_7_mm SEASON_7_mm if gender_7 == 2 , irr
